# Short-term Outcomes After Robot-Assisted vs Open Pancreaticoduodenectomy After the Learning Curve

**DOI:** 10.1001/jamasurg.2020.0021

**Published:** 2020-03-04

**Authors:** Yusheng Shi, Jiabin Jin, Weihua Qiu, Yuanchi Weng, Jian Wang, Shulin Zhao, Zhen Huo, Kai Qin, Yue Wang, Hao Chen, Xiaxing Deng, Chenghong Peng, Baiyong Shen

**Affiliations:** 1 Pancreatic Disease Center, Ruijin Hospital, Shanghai Jiaotong University School of Medicine, Shanghai, China; 2 Xinhua Hospital, Shanghai Jiaotong University School of Medicine, Shanghai, China

## Abstract

**Importance:**

Robot-assisted pancreaticoduodenectomy (RPD) has been reported to be safe and feasible. As a new technique, RPD has a learning curve similar to that of other types of minimally invasive pancreatic surgery such as laparoscopic pancreaticoduodenectomy. To our knowledge, no reports exist on the outcomes of open pancreaticoduodenectomy (OPD) and RPD after the learning curve.

**Objective:**

To analyze and evaluate the actual advantages of RPD.

**Design, Setting, and Participants:**

Between May 2010 and December 2018, 450 patients underwent RPD in the Shanghai Ruijin Hospital affiliated with Shanghai Jiaotong University in Shanghai, China, a high-volume pancreatic disease center. According to our previous study, an important flexion point in the learning curve is 250 cases. Data on the last 200 RPD cases were collected from January 2017 to December 2018. During that period, 634 patients underwent OPD. These patients were divided into 2 groups, and propensity score matching was used to minimize bias. The demographic data and operative outcomes were collected and analyzed. Analysis began May 2019.

**Exposures:**

Robot-assisted pancreaticoduodenectomy and OPD.

**Main Outcomes and Measures:**

The short-term operative outcomes of RPD and OPD.

**Results:**

After 1:1 matching, 187 cases of RPD and OPD were recorded. In the RPD group, 78 patients (41.7%) were women, and the mean (SD) age was 60.9 (11.4) years. In the OPD group, 80 patients (42.8%) were women, and the mean (SD) age was 60.1 (10.8) years. Robot-assisted pancreaticoduodenectomy had advantages in operative time (mean [SD], 279.7 [76.3] minutes vs 298.2 [78.3] minutes; *P* = .02), estimated blood loss (mean [SD], 297.3 [246.8] mL vs 415.2 [497.9] mL; *P* = .002), and postoperative length of hospital stay (mean [SD], 22.4 [16.7] days vs 26.1 [16.3] days; *P* = .03). However, there was no significant difference in the R0 resection rate and incidence rate of postoperative complications, such as postoperative pancreatic fistula, bile leak, and delayed gastric emptying. The incidence rates of postoperative bleeding and reoperation in the RPD group were similar to those in the OPD group, with no statistically significant difference.

**Conclusions and Relevance:**

After passing the learning curve, RPD had advantages in operative time and blood loss compared with OPD. There were no differences in postoperative complications such as postoperative pancreatic fistula, bile leak, and delayed gastric emptying. However, patients recovered more quickly after RPD than after OPD. A prospective randomized clinical trial is needed in the future to verify these results.

## Key Points

**Question:**

What are the actual advantages of robot-assisted pancreaticoduodenectomy (PD) after the learning curve?

**Findings:**

In this cohort study of 187 individuals, robot-assisted PD had advantages over open PD in operative time, estimated blood loss, and postoperative hospital stay after the learning curve.

**Meaning:**

The true advantages of robot-assisted PD could be revealed after passing the learning curve.

## Teaser

This cohort study assesses robot-assisted vs open pancreaticoduodenectomy after the learning curve.

## Introduction

Pancreaticoduodenectomy (PD) has been reported to have high morbidity and mortality because of the difficulty and complexity of the procedure.^1^ This approach is regarded as the most challenging type of pancreatic surgery and can cause great burden to the patient. The robot-assisted surgical system was first used in the last century, and recent reports about robot-assisted pancreatic surgery have been published in the literature. In 2003, Giulianotti et al^2^ reported the world’s first robot-assisted PD (RPD). Since then, an increasing number of centers have started offering RPD. Our pancreatic disease center first started performing RPD in 2009. Currently, we have completed more than 500 RPD procedures. We reported our first experience with RPD in 2015 and indicated that RPD is safe and feasible for selected patients.^3^ We also illustrated our learning curve (LC) with RPD through 450 cases.^4^ We discovered that the surgeon’s skills rapidly improved and reached a stable level after 250 cases. However, the effectiveness and feasibility of RPD remains controversial around the world.^5,6,7,8^ This study aimed to demonstrate the advantages of RPD over open PD (OPD) through a cohort of patients we analyzed after the illustrated LC.

## Methods

### Patient Selection and Study Design

In total, 450 patients underwent RPD from May 2010 to December 2018 in the Pancreatic Disease Center of the Shanghai Ruijin Hospital affiliated with Shanghai Jiaotong University School of Medicine in Shanghai, China. According to our previous results,^4^ case 250 was the flexion point. We analyzed the last 200 cases from the total cohort (February 2017 to December 2018). In the same period, 634 patients underwent OPD. A total of 45 patients with a history of abdominal surgery in the OPD group were excluded because of potential selection bias. All these surgeries were performed by the same group of surgeons (H.C., X.D., C.P., and B.S.). The demographic data and short-term operative outcomes were collected and analyzed. The mortality events were excluded from the postoperative length of stay (LOS) analysis. We used propensity score matching (PSM) to minimize bias from the patient selection process.

This study was approved by the institutional review board of Shanghai Ruijin Hospital. Informed consent was signed by every patient as the agreement for receiving the operation and the use of the data we collected before and after surgery.

### Matching

Propensity score matching was used to minimize bias from treatment selection when comparing 2 different treatments in the cohort study. It has been widely used in the medical literature.^9^ In this study, we collected the patient characteristics of our total cohort. From our experience and from previous reports, age, sex, body mass index (BMI), preoperative diabetes status, hemoglobin level, albumin level, American Society of Anesthesiologists score, and the pathological results were considered important factors associated with the short-term outcomes after OPD and RPD. In total, 200 cases of RPD and 634 cases of OPD were collected in our study. We calculated a propensity score for each patient through logistic regression modeling and then patients were matched 1:1, with the caliper width set as 0.01 for the SD. Standardized mean differences were estimated before and after matching to evaluate the balance and a value less than 0.1 was considered not significant between treatment groups. The patient demographic data were adjusted to almost the same level after matching.

The short-term outcomes such as operative time, estimated blood loss, complications including postoperative pancreatic fistula (POPF), bile leak, bleeding, reoperation, R0 resection, delayed gastric emptying, and postoperative LOS were recorded and analyzed. Long-term outcomes were not included in this study. Postoperative pancreatic fistula was diagnosed and classified according to the latest criteria by the International Study Group of Pancreatic Fistula.^10^

### Statistical Analysis

SPSS version 22.0 (IBM) was used for PSM and all calculations. GraphPad PRISM (GraphPad Software) was used for plotting. Continuous data are summarized as the means and SDs. For comparing the unmatched groups, the *t* test was used for continuous variables, and the χ^2^ test or Fisher exact test were used for categorical variables. For proportional outcome comparisons between RPD and OPD cohorts after PSM, the paired *t* test was used for continuous variables, and the McNemar test was used for binary variables. Two-sided *P* < .05 was considered statistically significant. Analysis began May 2019.

## Results

### Patient Characteristics

After PSM, 187 patients were included in each group. Our results indicated that no significant differences existed between the 2 groups in terms of age, sex, BMI, hemoglobin level, albumin level, American Society of Anesthesiologists score, tumor size, pathology, history of diabetes, smoking history, and alcohol consumption habits after matching. In the RPD group, 78 patients (41.7%) were women, and the mean (SD) age was 60.9 (11.4) years. A total of 98 patients (52.4%) had normal BMI levels, 148 patients (79.1%) had normal hemoglobin levels (≥11 g/dL in women and ≥12 g/dL in men [to convert to grams per liter, multiply by 10]), and 158 patients (84.5%) had normal albumin levels (≥3.5 g/dL [to convert to grams per liter, multiply by 10]). The mean (SD) tumor size was 2.7 (1.1) cm. Robot-assisted PD was performed in 63 cases (33.7%) of benign or low-grade malignant pancreatic or periampullary tumors, 86 cases (46.0%) of pancreatic adenocarcinoma or chronic pancreatitis, and 38 cases (20.3%) of malignant periampullary tumors. In the OPD group, 80 patients (42.8%) were women, and the mean (SD) age was 60.1 (10.8) years. A total of 98 patients (52.4%) had normal BMI levels. A total of 146 patients (78.1%) had normal hemoglobin levels, and 158 patients (84.5%) had normal albumin levels. The mean (SD) tumor size was 2.7 (1.3) cm. Open PD was performed in 57 cases (30.5%) of benign or low-grade malignant pancreatic or periampullary tumors, 101 cases (54.0%) of pancreatic adenocarcinoma or chronic pancreatitis, and 29 cases (15.5%) of malignant periampullary tumors. The patient characteristics are displayed in Table 1.

**Table 1.** Patient Characteristics Before and After Propensity Score Matching

Characteristic	Propensity Score Matching, No. (%)							
	Before				After			
	RPD (n = 200)	OPD (n = 634)	*P* Value	SMD	RPD (n = 187)	OPD (n = 187)	*P* Value	SMD
Age, mean (SD), y	59.4 (12.6)	62.7 (10.5)	<.001	0.285	60.9 (11.4)	60.1 (10.8)	.52	0.072
Sex								
Female	88 (44.0)	257 (40.5)	.39	0.071	78 (41.7)	80 (42.8)	.61	0.022
Male	112 (56.0)	377 (59.5)			109 (58.3)	107 (57.2)		
BMI								
<19	21 (10.5)	82 (12.9)	.01	0.202	19 (10.2)	24 (12.8)	.51	0.050
19-24	104 (52.0)	385 (60.7)			98 (52.4)	98 (52.4)		
≥24	75 (37.5)	167 (26.3)			70 (37.4)	65 (34.8)		
Hemoglobin level^a^								
Normal	40 (20.0)	179 (28.2)	.02	0.150	39 (20.9)	41 (21.9)	.90	0.017
Abnormal	160 (80.0)	455 (71.8)			148 (79.1)	146 (78.1)		
American Society of Anesthesiologists score								
1	64 (32.0)	190 (30.0)	.56	0.037	56 (29.9)	59 (31.6)	.88	0.042
2	128 (64.0)	407 (64.2)			123 (65.8)	119 (63.6)		
≥3	8 (4.0)	37 (5.8)			8 (4.3)	9 (4.8)		
Albumin level^b^								
Normal	29 (14.5)	152 (24.0)	.005	0.169	29 (15.5)	29 (15.5)	>.99	0
Abnormal	171 (85.5)	482 (76.0)			158 (84.5)	158 (84.5)		
Diabetes								
No	36 (18.0)	117 (18.5)	.89	0.010	36 (19.3)	35 (18.7)	>.99	0.010
Yes	164 (82.0)	517 (81.5)			151 (80.7)	152 (81.3)		
History of smoking								
No	152 (76.0)	491 (77.4)	.67	0.025	139 (74.3)	139 (74.3)	>.99	0
Yes	48 (24.0)	143 (22.6)			48 (25.7)	48 (25.7)		
History of alcohol use								
No	169 (84.5)	535 (84.4)	.97	0.002	156 (83.4)	152 (81.3)	.68	0.036
Yes	31 (15.5)	99 (15.6)			31 (16.6)	35 (18.7)		
Tumor size, mean (SD) cm	2.7 (1.1)	3.1 (1.5)	.002	0.304	2.7 (1.1)	2.7 (1.3)	.72	0
Pathology								
Benign or low-grade malignant tumors located at the pancreatic head and periampullary area^c^	76 (38.0)	82 (12.9)	<.001	0.403	63 (33.7)	57 (30.5)	.76	0.060
Malignant tumors located at the pancreatic head^d^ and chronic pancreatitis	96 (48.0)	396 (62.5)			96 (51.3)	101 (54.0)		
Malignant periampullary tumors^d^	28 (14.0)	156 (24.6)			28 (15.0)	29 (15.5)		

Abbreviations: BMI, body mass index (calculated as weight in kilograms divided by height in meters squared); OPD, open pancreaticoduodenectomy; RPD, robot-assisted pancreaticoduodenectomy; SMD, standardized mean difference.

^a^ Normal hemoglobin level is ≥11 g/dL in women and ≥12 g/dL in men (to convert to g/L, multiply by 10).

^b^ Normal albumin level is ≥3.5 g/dL (to convert to g/L, multiply by 10).

^c^ Lymph node clearance was not required.

^d^ Lymph node clearance was required.

### Operative Outcomes

From our data, RPD had advantages in operative time (mean [SD], 279.7 [76.3] vs 298.2 [78.3] minutes; 95% CI, −34.2293 to −2.7760; *P* = .02), estimated blood loss (mean [SD], 297.3 [246.8] vs 415.2 [497.9] mL; 95% CI, −197.8848 to −38.0510; *P* = .002), and postoperative length of hospital stay (mean [SD], 22.4 [16.7] vs 26.1 [16.3] days; 95% CI, −7.0837 to -0.3708; *P* = .03). The R0 resection was 5.3% (10 of 187 in RPD group) and 7.0% (13 of 187 in OPD group) (*P* = .68). The incidence rate of clinically relevant POPF in the RPD group was 10.2% (19 of 187), while the incidence rate of biochemical leakage was 4.8% (9 of 187). In the OPD group, the incidence rate of clinically relevant POPF was 14.4% (27 of 187), while the incidence rate of biochemical leakage was 1.1% (2 of 187). There was no statistically significant difference in POPF rate, R0 resection rate, or incidence rate of postoperative complications such as POPF, bile leak, and delayed gastric emptying. Additionally, there was no difference in the incidence rate of postoperative bleeding or reoperation. However, the incidence rate of abdominal infection was lower in RPD group (40 of 187 [21.4%] vs 64 of 187 [34.2%]; *P* = .008). The short-term postoperative outcomes are in Table 2.

**Table 2.** Short-Term Operative Outcomes of the 2 Groups

Outcome	Propensity Score Matching, No. (%)					
	Before			After		
	RPD (n = 200)	OPD (n = 634)	*P* Value	RPD (n = 187)	OPD (n = 187)	*P* Value
Operative time, mean (SD), min	278.2 (76.8)	301.3 (80.2)	<.001	279.7 (76.3)	298.2 (78.3)	.02
Estimated blood loss, mean (SD), mL	213.6 (173.0)	418.4 (398.9)	<.001	297.3 (246.8)	415.2 (497.9)	.002
R0 resection	190 (95.0)	577 (91.0)	.07	177 (94.7)	174 (93.0)	.68
Lymph node harvest	16.3 (6.5)	14.2 (9.4)	.02	16.6 (6.2)	15.8 (10.0)	.495
Biochemical leak and CR-POPF	28 (14.0)	79 (12.5)		28 (15.0)	29 (15.6)	
Biochemical leak	9 (4.5)	19 (3.0)	.57	9 (4.8)	2 (1.1)	>.99
CR-POPF	19 (9.5)	60 (9.5)	.07	19 (10.2)	27 (14.4)	.09
Grade B	12 (6.0)	19 (3.0)		12 (6.4)	14 (7.5)	
Grade C	7 (3.5)	41 (6.5)		7 (3.7)	13 (7.0)	
Bile leak	11 (5.5)	35 (5.5)	.99	10 (5.3)	10 (5.3)	>.99
Gastrojejunostomy leak	2 (1.0)	6 (0.1)	.95	2 (1.1)	2 (1.1)	>.99
Delayed gastric emptying	9 (4.5)	15 (2.4)	.12	9 (4.8)	5 (2.7)	.42
Infection	42 (21.0)	176 (27.8)	.06	40 (21.4)	64 (34.2)	.008
Postoperative bleeding	10 (5.0)	32 (5.0)	>.99	8 (4.3)	9 (4.8)	>.99
Reoperation	7 (3.5)	41 (6.5)	.12	7 (3.7)	13 (7.0)	.26
90-d mortality	5 (2.5)	15 (2.4)	.91	4 (2.1)	7 (3.7)	.47
Postoperative LOS,^a^ mean (SD), d	21.8 (16.5)	24.1 (14.9)	.07	22.4 (16.7)	26.1 (16.3)	.03

Abbreviations: CR-POPF, clinically relevant postoperative pancreatic fistula; LOS, length of stay; OPD, open pancreaticoduodenectomy; RPD, robot-assisted pancreaticoduodenectomy.

^a^ The cases of death were excluded in analyses of the postoperative LOS.

## Discussion

Pancreaticoduodenectomy is one of the most difficult types of pancreatic surgery because of the complexity of the procedure.^11^The robot-assisted surgical system has been launched for more than 20 years. However, owing to technique challenge, the first RPD was reported only about 15 years ago in 2003 by Giulianotti et al.^2^ In recent years, by rapid progress in robot-assisted pancreatic surgery, many centers have reported their initial experiences with RPD.^5,6,7,8^ In 2012, Lai et al^8^ reported 20 cases of RPD with a mean operative time of nearly 500 minutes, which is much longer than that of OPD (264.9 minutes). Meanwhile, no better postoperative complications could be observed in the RPD group than those who received OPD. In 2013, Zureikat et al^7^ reported their experience with 250 robot-assisted pancreatic surgeries, which was the largest sample size in the literature, to our knowledge. In their study, 132 RPD cases were included with a morbidity of nearly 62%. In 2015, our center reported our first experience with RPD,^3^ and the overall morbidity was 35%, which was similar to that of OPD. However, we observed that the operative time at the early stage of RPD was much longer than that of OPD (445 minutes vs 322 minutes; *P* < .001). Other than the rate of infection, the operative outcomes of RPD were not advantageous over those of OPD. Therefore, we could easily recognize that, in the early period, RPD did not have many advantages over OPD, and the main benefits of RPD were less surgical burden.

Different from the results in reports regarding the comparison of RPD and OPD,^12^ in this study, RPD had advantages in operative time, estimated blood loss, and postoperative hospital stay. We believed that the convincing LC should be established based on enough case numbers and surgical outcomes of RPD should be evaluated with those of OPD in the same LC period. There have been several reports about the LC of RPD. In 2016, Napoli et al^13^ collected 70 cases of RPD and found 2 phases of the LC with a flexion point at case 33. Zhang et al^14^ reported their LC in 2019 with a sample size of 100 cases. The authors also found 2 phases with a flexion point at case 40. Interestingly, Boone et al^15^ found 3 phases in their LC with a larger sample size of 200 patients: cases 80 and 140 were the 2 inflexion points. From 2009 to 2018, 450 RPD procedures were performed in our center. Similar to the results of Boone et al,^15^ we also found 3 phases, although the inflexion points in our study were cases 100 and 250. After 250 cases, the operative time and estimated blood loss could be stabilized, indicating that the surgeons understood the technique comprehensively. In our study, the surgical outcomes of RPD were better than those of OPD, which were performed in the same LC stage. Therefore, comparison between RPD and OPD should be scientifically accomplished along with the different LC stage.

Owing to the patient selection bias, in our baseline cohort, the operative time and estimated blood loss of RPD were significantly better than those of OPD. There were more cases of malignant pancreatic tumors and chronic pancreatitis in the OPD group. In those cases, surgery would be more difficult, causing longer operative time, more blood loss, and longer postoperative LOS. Therefore, PSM, which has been a popular statistical method in recent years, was used to reduce bias. Several reports have used PSM to compare these 2 procedures.^16,17^ The surgical and oncological outcomes of RPD have been proven, indicating that RPD is a feasible surgery for malignant tumors. In our study, after PSM, 187 patients were included in each group. Meanwhile, based on our above results, by stabilizing surgical skill after 250 cases, the comparisons between RPD and OPD are able to reflect the advantages of RPD more accurately. Per the results, RPD did have advantages in operative time, estimated blood loss, and postoperative LOS, which revealed that RPD could cause less surgical burden and provide faster recovery than OPD. The improvement in the RPD operative time was mainly because we optimized and modularized our surgical procedure. The modularized surgical protocol could be helpful not only in improving the safety of RPD but also in saving time during the preparation, docking, and operative manipulation. However, gaining experience could not lead to a reduction in morbidity and mortality. In our study, there looks like no improvement in the incidence rate of biochemical leakage and clinically relevant POPF in RPD, although *P* = .09 may be suggestive of a noteworthy difference. The clinical influence of pancreatic fistula may approach significance with more patients in our continuing study. For postoperative LOS, as there were several cases of death after PSM, there might be some potential bias.

In the RPD and OPD procedures, the maneuver of pancreaticojejunostomy (PJ) anastomoses was technically the same. Using 6-0/5-0 prolene for the inner layer suture, a duct-to-mucosa double layer anastomosis was performed. The outer layer suture with 3-0 prolene could reduce the tension of the reconstruction. The most remarkable disadvantage of RPD is that the surgeon lacks tactile feedback, which makes it difficult to judge the optimal tightness of the reconstruction. Therefore, a novel method, visual-tactile feedback, was developed, by which the tightness of PJ reconstruction can be visually evaluated according to the deformations of the pancreas and jejunum. By this method, the POPF rate in RPD could be apparently reduced in our previous study.^18^ At the early stage, the PJ in RPD was finished in 40 to 50 minutes. After the LC, the time for the PJ approximately decreased to 20 to 30 minutes, which was even shorter than that in OPD in some cases. Although the morbidity was similar, patients in the RPD group were ambulant more quickly than patients in the OPD group with shorter hospital stay. Since the imbalance of medical support level around the country, patients discharged with drainage tubes or requiring additional parenteral nutrition might not have enough rehabilitation. On the other hand, for postoperative emergencies, such as late-phase bleeding, the local hospitals did not have the ability to provide the appropriate care. Therefore, hospitalization in China was longer than that in Western countries. In this scenario, even with the same maneuver of PJ anastomoses, the less surgical burden in RPD would be helpful to decrease the hospitalization.

In recent years, minimally invasive pancreatic surgery has been accepted by an increasing number of centers. After comparing with recent reports of RPD with more than 50 cases (Table 3),^19,20,21^ we can see that the operative time was all longer than 300 minutes. However, in our opinion, the main reason could be that the surgeons were still passing the LC. After the LC, our operative time was approximately 240 minutes, which was even better than that of OPD. Meanwhile, our study indicated that a well-modularized surgical procedure would lead to a better and more stable operative outcome in overall morbidity, POPF rate, and mortality. Therefore, considering the complexity of the procedure, the mature surgical technique and postoperative management strategies were closely associated with case number and experience accumulation.

**Table 3.** Comparison Between Our Study and Other Centers

Source	Case No.	Mean (SD)		%		Postoperative LOS, Mean (SD), d	Mortality, %
		Operative Time, min	Estimated Blood Loss, mL	Overall Morbidity	POPF Rate		
Zhang et al,^14^ 2019	100	357.9 (93.3)	171.1 (144.5)	58	24	18 (13.46)	7.5
Kim et al,^19^ 2018	51	335.6 (69.4)	361.2 (219.6)	15.7	5.9^a^	10.6 (8.3)	2
Guerra et al,^20^ 2019	59	515 (390-720)^b^	150 (300-900)^b^	37.3	16.9	9 (5-110)	3.3
Wang et al,^17^ 2018	118	433 (131)	177 (182)	44.1	NA	27 (16)	0
Nassour et al,^21^ 2017	193	422 (399)	NA	54.9	20.8	10.7 (8	1
Napoli et al,^13^ 2016	70	522.1 (98.0)	NA	75.7	35.7	23.2 (14.3)	NA
Boone et al,^15^ 2015	200	483 (113)	250 (150-500)^b^	67.5	17	9 (NA)	3.3
Zureikat et al,^7^ 2013	132	527 (NA)	NA	62	7.4	10 (NA)	3.8
Present study							
RPD post LC	200	278.2 (76.8)	213.4 (173.0)	36.5	15	21.8 (16.5)	2.5

Abbreviations: LC, learning curve; LOS, length of stay; NA, not applicable; POPF, postoperative pancreatic fistula; RPD, robot-assisted pancreaticoduodenectomy.

^a^ Only grade B and C POPF.

^b^ Median (interquartile range) is reported.

### Limitations

The limitations to this study were the bias associated with patient selection and the retrospective nature of the research. Further randomized clinical trials should be designed.

## Conclusions

After the LC, the operative outcomes of RPD were better than those of OPD, including operative time, estimated blood loss, and postoperative hospital stay. Because this study is a retrospective study, we used the PSM method to reduce the potential bias. Our ongoing randomized clinical studies would further verify the advantages of robot-assisted pancreatic surgery.
